# The association of hyponatremia and early postoperative complications in aseptic revision total shoulder arthroplasty

**DOI:** 10.1007/s00590-024-04054-x

**Published:** 2024-08-12

**Authors:** Steven H. Liu, Allen Bramian, Rachel A. Loyst, Kevin Kashanchi, Edward D. Wang

**Affiliations:** 1 Department of Orthopaedics, Keck Medicine of University of Southern California, 1540 Alcazar Street CHP 207, Los Angeles, CA 90089-9007 USA; 2 Department of Orthopaedics, Stony Brook University, Stony Brook, NY USA

**Keywords:** Revision total shoulder arthroplasty, Total shoulder arthroplasty, Hyponatremia sodium, Complications

## Abstract

**Purpose:**

This study investigates the association between preoperative serum sodium levels and 30-day postoperative complications following aseptic revision total shoulder arthroplasty (TSA).

**Methods:**

The American College of Surgeons National Surgical Quality Improvement Program database was queried for all patients who underwent aseptic revision TSA from 2015 to 2022. The study population was divided into two groups based on preoperative serum sodium levels: eunatremia (135–144 mEq/L) and hyponatremia (< 135 mEq/L). Logistic regression analysis was performed to investigate the relationship between hyponatremia and early postoperative complications.

**Results:**

Compared to eunatremia, hyponatremia was independently associated with a significantly greater likelihood of experiencing any complication (odds ratio [OR] 1.65, 95% confidence interval [CI] 1.14–2.40; *P* = .008), blood transfusions (OR 2.45, 95% CI 1.24–4.83; *P* = .010), unplanned reoperation (OR 2.27, 95% CI 1.07–4.79; *P* = .032), and length of stay > 2 days (OR 1.63, 95% CI 1.09–2.45; *P* = .017).

**Conclusion:**

Hyponatremia was associated with a greater rate of early postoperative complications following noninfectious revision TSA. This study sheds light on the role of preoperative hyponatremia as a risk factor for postoperative complications and may help surgeons better select surgical candidates and improve surgical outcomes in the setting of revision TSA.

## Introduction

Revision total shoulder arthroplasty is often indicated to treat complications associated with primary total shoulder arthroplasty (TSA) [1]. Recent studies have reported a concurrent rise in primary and revision TSA [2–4]. A recent systematic review and meta-analysis reported high complication rates following revision TSA [5]. The rise in revision TSA volume and associated postoperative complications warrants investigation into preoperative factors that may serve as predictors of postoperative complications following revision TSA.

Hyponatremia, defined as a serum sodium level below 135 mEq/L, is an electrolyte abnormality that leads to symptoms ranging from headache and nausea to seizures and coma. Studies have demonstrated an association between preoperative hyponatremia and morbidity and mortality following both orthopedic and non-orthopedic surgery [6–8]. A study investigating the relationship between preoperative hyponatremia and postoperative complications following aseptic revision hip and knee arthroplasty revealed hyponatremia to be an independent risk factor for multiple postoperative complications [9]. Another study reported associations between preoperative hyponatremia and postoperative complications in primary TSA [8].

Hyponatremia as a preoperative risk factor may exacerbate the already relatively high complication rates associated with revision TSA. Yet, there is a paucity of literature studying the effects of hyponatremia in the setting of revision TSA. This study aims to determine the relationship between preoperative hyponatremia and 30-day postoperative complications following revision TSA. We hypothesized that hyponatremia would be associated with higher rates of 30-day postoperative complications following revision TSA.

## Materials and methods

We queried the American College of Surgeons National Surgical Quality Improvement Program (ACS-NSQIP) database for all patients who underwent revision TSA from 2015 to 2022. This study was exempt from approval by our University’s Institutional Review Board as the NSQIP database is fully deidentified. Data in the NSQIP database are gathered from over 600 hospitals in the USA by trained surgical clinical reviewers [10].

**Fig. 1:**
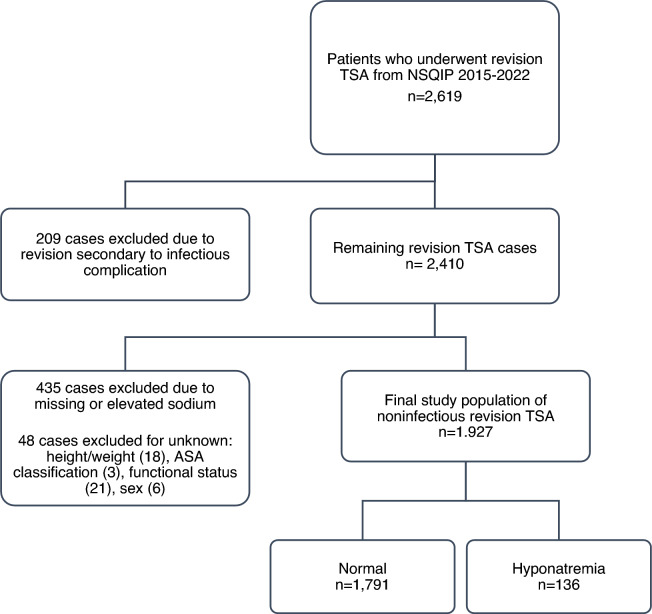
Case selection schematic detailing patient selection process for revision total shoulder arthroplasty (TSA) from 2015 to 2022. TSA, total shoulder arthroplasty; *NSQIP*, National Surgical Quality Improvement Program; ASA, American Society of Anesthesiologists

The *Current Procedural Terminology* (CPT) codes 23,473 and 23,474 were used to identify 2,619 patients who underwent revision TSA from 2015 to 2022 (Fig. 1). The NSQIP database excludes all cases for patients younger than 18 years of age and those with primary admission related to trauma. Initially, 209 revision TSA cases were excluded as revision was performed due to an infectious primary etiology. We opted to exclude revisions performed for an infectious indication because the NSQIP database lacks details regarding the nature of the infection (i.e., acute vs. chronic), which may significantly impact postoperative complication rates. Next, 435 cases were excluded due to either missing preoperative sodium measurements or sodium measurements not belonging in the eunatremia or hyponatremia ranges. Additionally, 48 cases were excluded for unknown information relating to height/weight, the American Society of Anesthesiologists (ASA) classification, functional health status, or sex. The final patient population included in the study after applying exclusion criteria was 1,927. These cases were then separated based on serum sodium levels into eunatremia (135–144 mEq/L) and hyponatremia (< 135 mEq/L) cohorts: 1,791 patients in eunatremia and 136 patients in hyponatremia.

Variables collected in this study included patient demographics, comorbidities, surgical characteristics, and 30-day postoperative complication data. Patient demographics included sex, age, body mass index (BMI), functional status, ASA classification, smoking status, and preoperative steroid use. Preoperative comorbidities included congestive heart failure (CHF), diabetes mellitus, hypertension, severe chronic obstructive pulmonary disease (COPD), bleeding disorders, and disseminated cancer. Perioperative factors included total operation time. Thirty-day complications included the following: sepsis, septic shock, pneumonia, unplanned reintubation, urinary tract infection (UTI), cardiac arrest or myocardial infarction (MI), stroke, blood transfusions, deep-vein thrombosis (DVT), pulmonary embolism (PE), on ventilator > 48 h, surgical space infection (SSI), wound dehiscence, acute renal failure, *Clostridioides difficile*** (***C. diff*) infection, nonhome discharge, readmission, unplanned reoperation, periprosthetic fracture, length of stay (LOS) > 2 days, and mortality.

Statistical analyses were performed using Python version 3.8 with the Statsmodels Python package. Bivariate logistic regression was used to compare patient demographics and comorbidities between the two groups. Multivariate logistic regression, adjusted for all patient demographics and comorbidities significantly associated with hyponatremia, was used to identify significant independent associations between hyponatremia and postoperative complications. Odds ratios (ORs) were reported with 95% confidence intervals (CI). The level of statistical significance was set at *P* < 0.05.

## Results

Compared to eunatremia, hyponatremia was significantly associated with BMI less than 24.9 kg/m^2^ *(P* = 0.004), dependent functional status prior to surgery *(P* = 0.000), and comorbid hypertension (*P* = 0.006) (Table 1).

**Table 1:** Demographics and comorbidities of patients with eunatremia and hyponatremia. Bold *P* values indicate statistical significance with *P* < .05

	Eunatremia (135–144 mEq/L)	Hyponatremia (< 135 mEq/L)	
Characteristics	Number (%)	Number (%)	*P* value
Overall	1791 (100.0)	136 (100.0)	
Sex			0.750
Female	936 (52.3)	73 (53.7)	
Male	855 (47.7)	63 (46.3)	
Age			0.339
18–39	12 (0.7)	2 (1.5)	
40–59	297 (16.6)	18 (13.2)	
60–79	1284 (71.7)	97 (71.3)	
≥ 80	198 (11.1)	19 (14.0)	
BMI (kg/m^2^)			**0.004**
< 18.5	7 (0.4)	1 (0.7)	
18.5–24.9	270 (15.1)	36 (26.5)	
25–29.9	548 (30.6)	36 (26.5)	
≥ 30	966 (53.9)	63 (46.3)	
Functional status prior to surgery			**0.000**
Dependent	52 (2.9)	13 (9.6)	
Independent	1739 (97.1)	123 (90.4)	
ASA classification			0.056
≤ 2	647 (36.1)	38 (27.9)	
≥ 3	1144 (63.9)	98 (72.1)	
Smoker			0.894
No	1600 (89.3)	121 (89.0)	
Yes	191 (10.7)	15 (11.0)	
Steroid use			0.223
No	1678 (93.7)	131 (96.3)	
Yes	113 (6.3)	5 (3.7)	
Comorbidities			
CHF	32 (1.8)	3 (2.2)	0.725
Diabetes	0 (0.0)	0 (0.0)	0.903
Hypertension	1203 (67.2)	107 (78.7)	**0.006**
COPD	137 (7.6)	11 (8.1)	0.853
Bleeding disorder	61 (3.4)	6 (4.4)	0.538
Disseminated cancer	5 (0.3)	0 (0.0)	0.995
Total operation time (minutes)			0.383
0–79	396 (22.1)	31 (22.8)	
80–128	709 (39.6)	60 (44.1)	
≥ 129	686 (38.3)	45 (33.1)	

BMI, body mass index; ASA, American Society of Anesthesiologists; CHF, congestive heart failure; COPD, chronic obstructive pulmonary disease

Compared to eunatremia, hyponatremia was significantly associated with a greater likelihood of experiencing any complication *(P* = 0.000), blood transfusions *(P* = 0.001), nonhome discharge *(P* = 0.004), unplanned reoperation *(P* = 0.021), and LOS > 2 days *(P* = 0.001) (Table 2).

**Table 2:** Bivariate analysis of 30-day postoperative complications in patients with eunatremia and hyponatremia. Bold *P* values indicate statistical significance with *P* < .05

	Eunatremia (135–144 mEq/L)	Hyponatremia (< 135 mEq/L)	
Complications	Number (%)	Number (%)	*P* value
Any complication	461 (25.7)	54 (39.7)	**0.000**
Sepsis	11 (0.6)	1 (0.7)	0.863
Septic shock	1 (0.1)	0 (0.0)	0.851
Pneumonia	3 (0.2)	0 (0.0)	0.988
Unplanned reintubation	3 (0.2)	0 (0.0)	0.988
UTI	12 (0.7)	1 (0.7)	0.929
Cardiac arrest or MI	8 (0.4)	0 (0.0)	0.998
Stroke	0 (0.0)	0 (0.0)	–
Blood transfusions	54 (3.0)	12 (8.8)	**0.001**
DVT	10 (0.6)	0 (0.0)	1.000
PE	10 (0.6)	1 (0.7)	0.792
On ventilator > 48 h	1 (0.1)	0 (0.0)	0.851
SSI	47 (2.6)	5 (3.7)	0.468
Wound dehiscence	4 (0.2)	0 (0.0)	0.999
Acute renal failure	3 (0.2)	0 (0.0)	0.988
*Clostridioides difficile* infection	2 (0.1)	0 (0.0)	0.999
Nonhome discharge	118 (6.6)	18 (13.2)	**0.004**
Readmission	80 (4.5)	8 (5.9)	0.447
Unplanned reoperation	52 (2.9)	9 (6.6)	**0.021**
Periprosthetic fracture	0 (0.0)	0 (0.0)	–
LOS > 2 days	317 (17.7)	40 (29.4)	**0.001**
Mortality	2 (0.1)	0 (0.0)	0.999

UTI, urinary tract infection; MI, myocardial infarction; DVT, deep-vein thrombosis; PE, pulmonary embolism; SSI, surgical site infection; LOS, length of stay

After controlling for all associated patient demographic and comorbid factors (BMI, functional status, and comorbid hypertension), an adjusted multivariate regression analysis was conducted. Compared to eunatremia, hyponatremia was independently associated with a significantly greater likelihood of experiencing any complication (odds ratio [OR] 1.65, 95% confidence interval [CI] 1.14–2.40; *P* = 0.008), blood transfusions (OR 2.45, 95% CI 1.24–4.83; *P* = 0.010), unplanned reoperation (OR 2.27, 95% CI 1.07–4.79; *P* = 0.032), and LOS > 2 days (OR 1.63, 95% CI 1.09–2.45; *P* = 0.017). (Table 3).

**Table 3:** Multivariate analysis of 30-day postoperative complications in patients with eunatremia and hyponatremia. Bold *P* values indicate statistical significance with *P* < .05

Complications	OR, P value, (95% CI)
Any complication	1.65, **0.008**, (1.14–2.40)
Blood transfusions	2.45, **0.010**, (1.24–4.83)
Nonhome discharge	1.66, 0.074, (0.95–2.90)
Unplanned reoperation	2.27, **0.032**, (1.07–4.79)
LOS > 2 days	1.63, **0.017**, (1.09–2.45)

LOS, length of stay

## Discussion

This study investigated hyponatremia as a risk factor for 30-day complications following revision TSA. Investigation of 1,927 patients from the NSQIP database who underwent revision TSA between the years of 2015 and 2022 revealed hyponatremia to be significantly associated with a higher rate of 30-day postoperative complications. Relative to eunatremia, hyponatremia was found to be an independently significant predictor of any complication, blood transfusions, unplanned reoperation, and postoperative hospital stay lasting longer than 2 days following revision TSA.

Hyponatremia is the most common electrolyte abnormality encountered in clinical practice and represents a disruption of water balance resulting in excess water relative to total body sodium content [11]. Sodium is the primary cation in the extracellular fluid and plays a role in numerous physiological processes [12]. As such, disruptions in serum sodium levels may lead to many symptoms ranging from headache to seizures and may ultimately lead to death [13]. Hyponatremia can lead to cellular dysfunction by causing osmotic imbalances, resulting in intracellular swelling and edema. In the context of surgery, tissue edema can compromise wound healing and increase the risk of infection. Additionally, hyponatremia is often associated with other electrolyte imbalances such as hypokalemia. These imbalances can predispose patients to cardiac arrhythmias, which can complicate the perioperative and postoperative periods. Although the full impact of hyponatremia is not entirely understood, healthcare providers recognize the crucial importance of detecting and correcting hyponatremia before revision total shoulder arthroplasty.

Hyponatremia, defined as a serum sodium concentration below 135 mEq/L, has been identified as a preoperative prognostic marker for perioperative 30-day morbidity and mortality in a wide range of surgical specialties [6]. Furthermore, studies have linked preoperative hyponatremia to 30-day postoperative complications following orthopedic procedures, including lumbar, shoulder, hip, and knee surgery [7–9, 14, 15]. This study provides evidence for preoperative hyponatremia as an independent predictor of 30-day postoperative complications following revision TSA.

Reported rates of blood transfusions following primary TSA vary, with studies reporting rates of up to 6.7% for primary TSA and up to 31% for revision TSA [16, 17]. Our study reports a greater than twofold increase in the risk of receiving blood transfusions in the hyponatremic group compared to the eunatremic group following revision TSA. In addition, 8.8% of the hyponatremic group received blood transfusions following revision TSA as compared to 3.0% in the eunatremic group. In general, revision TSA has an inherently higher risk of requiring blood transfusions and hyponatremia may exacerbate the need for blood transfusions in an already vulnerable population [17].

Relative to eunatremia, hyponatremia was associated with a greater than twofold increase in the risk of undergoing unplanned reoperation following revision TSA. A study on serious adverse events following revision TSA found that 41% of the reported perioperative complications occurred during an unplanned reoperation [3], highlighting the notion that a noteworthy proportion of all serious adverse events related to revision TSA occur during reoperation. Since preoperative hyponatremia is a significant predictor of unplanned reoperation in revision TSA patients, hyponatremia is a laboratory abnormality that should be factored into a patient’s preoperative risk determination to avoid further surgery and precipitously worse outcomes.

Nevertheless, comorbidities such as CHF and low body weight have been reported as predictors of increased length of stay following orthopedic procedures [18]. Our study, which controlled for differences in patient BMI, revealed that hyponatremia was associated with a greater than 1.5-fold likelihood of experiencing a hospital stay lasting greater than 2 days. Therefore, hyponatremia may have utility as a prognostic indicator for identifying cases at risk of prolonged hospital stays with the goal of decreasing surgical costs, increasing patient satisfaction, and decreasing nosocomial infections.

In the context of major surgery, preoperative hyponatremia is a prognostic marker for perioperative 30-day morbidity and mortality [6]. Specifically, it is associated with major morbidity and prolonged hospitalization in spine surgery, as well as SSIs, blood transfusions, pneumonia, sepsis, extended length of stay, wound, pulmonary, and infectious complications in aseptic revision hip and knee arthroplasty [7, 9]. Furthermore, preoperative hyponatremia has been associated with reoperation and prolonged hospitalization in total knee arthroplasty [19]. A study investigating hyponatremia in TSA reported similar associations between hyponatremia and major morbidity, prolonged length of stay, nonhome discharge, and readmission [8]. Altogether, the findings of this study align with preexisting orthopedic literature and extend our understanding of preoperative hyponatremia as a risk factor for early postoperative complications to the setting of revision TSA.

Postoperative care protocols, such as early mobilization, pain management, infection prevention measures (e.g., antibiotic prophylaxis and wound care), and as this study emphasized, screening for hyponatremia, are crucial for improving surgical outcomes. Identifying and correcting hyponatremia preoperatively can prevent related complications like seizures, confusion, and delayed recovery. These protocols help reduce the risk of complications like deep-vein thrombosis, pulmonary embolism, and surgical site infections, while also enhancing recovery times and overall patient satisfaction. Effective postoperative care, including proactive management of hyponatremia, can significantly decrease morbidity and mortality, leading to better long-term health outcomes for patients.

Limitations to this study include use of the NSQIP database which includes information that is limited to a postoperative window of 30 days. As such, we are unable to report on longer-term complications associated with preoperative hyponatremia. In addition, variables including surgeon experience and the center at which procedures were performed are not available. Indications for revision TSA are not included in this database. Therefore, cases may not be stratified based on the risk associated with varying indications. Furthermore, laboratory artifact may falsely indicate hyponatremia as is the case in pseudohyponatremia. It is unknown whether cases of pseudohyponatremia were erroneously included in this database and thus used in our study. Despite these limitations, our study provides utility in providing evidence for hyponatremia as a risk factor for 30-day postoperative complications following revision TSA.

## Conclusion

Our study identified preoperative hyponatremia as an independent predictor of 30-day postoperative complications following revision TlSA. As the utilization of primary and revision TSA continues to rise, preoperative laboratory values, including serum sodium levels, may offer benefit in risk stratification. By recognizing the risks associated with preoperative hyponatremia, surgeons may be better equipped to identify at risk surgical candidates and potentially correct modifiable preoperative risk factors.
